# Physiological response and drought resistance evaluation of *Gleditsia sinensis* seedlings under drought-rehydration state

**DOI:** 10.1038/s41598-023-45394-8

**Published:** 2023-11-15

**Authors:** Fuhua Liu, Yang Zhao, Xiurong Wang, Biao Wang, Feng Xiao, Kequan He

**Affiliations:** 1 Institute for Forest Resources and Environment of Guizhou, College of Forestry, Guizhou University, Guiyang, 550025 Guizhou China; 2 The State-Owned Forest Farm of Dushan County, Dushan, 558200 Guizhou China

**Keywords:** Physiology, Plant sciences

## Abstract

*G. sinensis* is a crucial tree species in China, possessing important economic and ecological value, and having a wide geographical distribution. *G. sinensis* seedlings is highly vulnerable to the drought-rehydration-drought cycle during their growth, and there is a lack of quantitative and systematic research on the physiological mechanisms of drought resistance and rehydration in *G. sinensis*. There is also a lack of good drought-resistant families and reliable methods for evaluating drought resistance, which severely hinders the selection and promotion of drought-resistant *G. sinensis* families and the industry's development. Therefore, this study selection 58 families seedlings of *G. sinensis * to drought stress and rehydration using an artificial simulated water control method in potted seedlings. The aim was to compare the effects of different levels of drought and rehydration on the growth and physiological indices of seedlings from different families. Identification of drought-resistant families and dependable drought related indices and techniques, the explanation of divergence in drought stress effects on various drought-resistant seedlings and the mechanisms underpinning growth and physiological responses, and the provision of theoretical reference for *G. sinensis* drought-resistant variety selection and cultivation. The Drought Resistance Index (DRI) served as the primary indicator, supplemented by growth, leaf morphology, and photosynthetic physiological indicators, to thoroughly assess and identify five distinct drought tolerant taxa while also selecting five representative families. Soluble protein (SP), proline (Pro), and malondialdehyde (MDA) contents, as well as the activities of catalase (CAT), peroxidase (POD) and superoxide dismutase (SOD) in seedlings from the five families, increased as the degree of drought intensified. The highest values were appeared during periods of severe drought, and gradually decreased after subsequent rehydration. Principal component analysis (PCA) revealed MDA and soluble sugars (SS) as the primary predictors of drought and rehydration response in *G. sinensis* seedlings respectively. Changes in osmoregulatory substance content and increased antioxidant enzyme activity may be crucial for responding to drought tolerance mechanisms. Leaf morphological indicators, seedling height, soil plant analysis development (SPAD) value, photosynthetic indicators, and MDA are dependable parameters for assessing the drought tolerance of *G. sinensis* seedlings. When assessing the drought-resistance of seedlings using physiological indicators such as photosynthesis, a comprehensive analysis should incorporate multiple indicators and methods. This evaluation approach could serve as a reference for screening exceptional drought-resistant families of *G. sinensis*.

## Introduction

Dramatic changes in global climate and precipitation patterns have worsened the pre-existing unequal allocation of water resources, resulting in drought conditions for numerous plants. Drought plays a crucial role in constraining the development of agroforestry, with decrement in crop yields due to drought surpassing that of other environmental factors combined^1,2^. Guizhou Province is one of the regions with the most severe karst landscapes in China^3^. Owing to significant water infiltration is severe and the special geographical environment leads to frequent temporary droughts, which to a certain extent limits the development of agroforestry. Drought stress has important effects on plant growth, development and physiology, mainly in terms of water redistribution in various parts, damage to membranes and nuclei, inhibition of leaf and root growth, reduced photosynthesis, excessive production of reactive oxygen species (ROS) and reduced osmotic potential. When plants were subjected to drought stress they accelerated the abnormal yellowing and shedding of leaves^4^. Previous researchers assessed the drought resistance of *Brassica napu*s L and five tree species using plant leaves affected by disasters and screened for high resistance (HR) and highly susceptible (HS) materials^5,6^. Drought resistance of plants could also be evaluated using photosynthetic parameters^7^. *Aronia melanocarpa*^8^ has increased MDA content and antioxidant enzyme activity with increased drought, and changes in its physiological indicators could also be used to evaluate drought resistance*.* Therefore, when evaluating the drought resistance of plants and screening for drought-resistant families, a comprehensive evaluation and analysis can be carried out in conjunction with plant morphology, photosynthesis and physiological and biochemical characteristics.

*G. sinensis* is an important ecological and economic tree species, widely distributed in China, and is one of the characteristic forestry species that Guizhou Province focuses on developing. The growth of *G. sinensis* is susceptible to drought due to its special geographical environment, and in its natural state is prone to a recurring pattern of drought-rehydration-drought cycles. Plants respond to drought through the reduction of water loss pathways and accumulation of osmoregulatory substances that decrease plant cell osmotic potential, which consequently enhanced water absorption capacity^9^. In 1-year-old *G. sinensis* seedlings, with prolonged drought, leaf water content and water potential showed a decreasing trend, Pro content showed a gradual increase, leaf SS content showed an increasing-decreasing-increasing trend, transpiration rate (Tr), stomatal conductance(Cs) continued to decrease, and water use efficiency continued to increase, whereas Pn first increased and then decreased^10^. Drought tolerance of seedlings from *G. microphylla* and *G. sinensis* were assessed using leaf chlorophyll (Chl) and water content^11^, Pro, SS and SP, and MDA content, CAT, POD^12^, and SOD activity, growth conditions, and stem and leaf wilting^13^. The results showed that both plants had a higher drought resistance, and seedlings increased osmotic regulator levels and antioxidant enzyme activity to improve tolerance. However, achieving accurate and uniform evaluation using various methods was difficult. Cluster analysis, Principal Component Analysis (PCA), correlation analysis, grey correlation degree analysis, drought tolerance metrics, weighted drought tolerance index and other methods have been jointly analysed to identify and screen key drought tolerance indices in multiple single traits. This study was conducted among various crop varieties, including citrus^14^, tea chrysanthemum^15^, *Hordeum vulgare* L ^16^ and maize^17^. Currently, many researches on *G. sinensis* mainly concentrates on seedling propagation technology and seedling physiological resistance^18–20^. However, there is a lack of quantitative systematic research on the physiological mechanisms of drought resistance and rehydration in different drought-resistant families of *G. sinensis* seedlings, coupled with a deficiency of accurate and uniform evaluation methods. This limitation has hampered the selection of drought-resistant families of *G. sinensis* for breeding, cultivation, promotion, and development of the industry. We artificially simulated the control of water in potted seedlings to subject *G. sinensis* seedlings to drought and rehydration. We compared the growth indices, leaf morphology, photosynthesis, and physiological indices among different drought-tolerant strains of *G. sinensis* to demonstrate differences in growth and physiology between drought and rehydration and the response mechanisms to drought stress in seedlings from different drought-tolerant *G. sinensis* families. Screening for drought-resistance indices to provide a theoretical reference for selecting and cultivating drought-resistant varieties of *G. sinensis*.

## Materials and methods

### Plant materials and conditions of cultivation

The materials comprised 58 high-quality seeds belonging to semi-sibling lineages of *G. sinensis*, collected during prior research on the germplasm resources of wild *G. sinensis* in Guizhou Province. These seeds were sourced from 21 counties and districts (“Appendix 1”)^21^. Seeds harvested, dried, and then refrigerated at 4 °C. They were then germinated and sown to produce seedlings in the spring time. The seeds were immersed in 98% concentrated sulphuric acid for 50 min, followed by rinsing with water and soaking in warm water at 38 °C until they were completely swollen and absorbed. The nursery soil underwent treatment with 0.1 percent potassium permanganate. The soil ratio used was humus: nutrient soil = 1:1. The pH of the potting soil was 7.53, with an average soil capacity of 1.25 g/cm^3^. The total nitrogen content was measured to be 10.84 g/kg, while the total phosphorus content was found to be 0.97 g/kg. The total potassium content was determined to be 3.36 g/kg. Additionally, the organic matter content was measured to be 13.98 g/kg. Seedling pots measuring 24 cm in height and 20 cm in diameter were utilized to accommodate each seedling, with each pot being loaded with 80% of its volume in soil from the nursery. One seedling was planted per pot. During the test period, temperatures fluctuated between 15.8 and 27.8 °C, while humidity levels ranged from 65 to 84%. The seedlings in pots were regularly tended to in a greenhouse located at the School of Forestry, Guizhou University. The greenhouse was draped with black shade netting, to provide about 60% shading. An illuminance meter (testo 540, Germany) was used to measure the illuminance at 13:00 on a clear day to be 2413 lux.

The seeds of *G. sinensis* were collected in this study with aid from the corresponding personnel of the Forestry and Grassland Bureau of Guizhou Province. Some wild plant seeds were collected after obtaining permission through friendly communication and consultation with the plant owners, without any conflict of interest. The collection of plant material and all experiments were performed following relevant institutional, national, and international guidelines and legislation.

### Methods

Seedlings were chosen for a natural drought treatment following five months of growth. The soil moisture was measured using a soil moisture meter (Delta-T, UK) and averaged from three readings. The test materials were thoroughly irrigated for three consecutive days prior to treatment, until water leaked out of the trays at the base of the pots to guarantee that the soil moisture level was uniform in each pot. This was considered the saturated soil moisture, which was between 30 and 35% soil moisture. The control group comprised a relative soil water content of 75–80%. Three drought gradients were established: mild drought (with a relative soil water content of 55–60%-expressed as a percentage of the maximum water-holding capacity of the soil); and severe drought (with a relative soil water content of 30–35%)^22^. The duration of natural drought was ascertained by reducing the water to the point where the water content lay between drought zones. The water content was measured using a soil moisture meter and the soil water content aimed to reach mild drought (7 continuous droughts) and severe drought (continuous droughts) conditions while using the control (relative water content of the soil ranging from 75 to 80%) as the control (0 d). The test for rehydration was carried out right after the drought test, during which the amount of water was gauged by a soil moisture meter to restore the relative soil moisture of the seedlings being tested to the control level. The degree of drought damage in the leaves of five seedling families gradually deepened with the prolongation of drought stress. Under severe drought stress, seedlings of HR and medium resistance (MR) families experienced slight harm, with a few leaves wilting and discoloring. Seedlings of low resistance (LR) and low susceptibility (LS) families were moderately affected, with nearly half of their leaves wilting and yellowing. However, seedlings of HS families were severely impacted, with a significant number of leaves wilting, curling up, and crumpling, leading to defoliation, and even some plant fatalities. Leaf morphology changed less after 1 d of rehydration, with leaves beginning to spread. After 3 d of rehydration, leaf continued to spread and fill with water, and the leaves of seedlings from HR and MR families began to change from yellow to green.

### Methods for determining morphological and photosynthetic indicators

Thirty plants from each family were chosen randomly from both the control and severe drought treatment groups to measure seedling height, diameter, and photosynthetic physiological indices. The SPAD values of the third leaf below the top of the plant were obtained via a SPAD device (502Plus, SAIYASI, Japan). The photosynthetic indices of the 3rd, 4th and 5th leaves below the top of the plant were collected using a portable LI6400 photosynthesis meter (LI-COR, USA) during sunny hours from 9am until 11am. Pn, Cs, intercellular CO_2_ concentration (Ci), and Tr were measured and recorded. The calculation and grading of the DRI was based on the leaf count of *G. sinensis* following 14 d drought period. Table 1 provides the grading criteria^6^. The greater the index value, the higher the plant's ability to withstand drought.

**Table 1:** Grading criteria for drought damage survey of *G. sinensis* leaves.

Grade	Drought symptoms
0	No obvious drought stress symptoms
1	Lightly affected, few leaves wilt and turn yellow
2	Moderate damage, about 1/2 of the leaves turning yellow and drying out
3	Severe damage, total leaf yellowing, massive defoliation

### Measurement of physiological indicators

The central section of the plant stem, intact and pest-free leaves were chosen from 0 d of drought, 7 d of drought, 14 d of drought, 1 d of rehydration, and 3 d of rehydration, and were washed with distilled water, then were frozen in liquid nitrogen after removing the water on the surface of the leaves, and stored in a refrigerator at − 80 ℃. Referring to Li Hesheng's method^23^, SS content was measured using the anthrone colourimetry. SP content was measured using the Bradford method^24^; Pro content was measured using the acidic ninhydrin colourimetric method^25^; MDA content was measured using the thiobarbituric acid method^26^; CAT enzyme activity was measured using the UV spectrophotometry ^27^; SOD enzyme activity was measured using the nitroblue tetrazolium method^28^; and POD enzyme activity was measured using the guaiacol method^29^.

### Data analysis

The data obtained were statistically analysed using Excel 2019 and IBM SPSS Statistics 26 and plotted using Origin 2022. Significance of differences between two sets of data were analysed using a t-test. For multiple comparisons of experimental data from various families at different drought times, the LSD method was employed (*p* < 0. 05), where the main formulas applied were as follows:

$$\varepsilon {\text{i}} = {{\left( {{\text{min}}_{{\text{i}}} {\text{min}}_{{\text{m}}} |{\text{A}}_{{\text{m}}} - {\text{B}}_{{{\text{im}}}} \left| { + \uprho {\text{max}}_{{\text{i}}} {\text{max}}_{{\text{m}}} } \right|{\text{A}}_{{\text{m}}} - {\text{B}}_{{{\text{im}}}} } \right)} \mathord{\left/ {\vphantom {{\left( {{\text{min}}_{{\text{i}}} {\text{min}}_{{\text{m}}} |{\text{A}}_{{\text{m}}} - {\text{B}}_{{{\text{im}}}} \left| { + \rho {\text{max}}_{{\text{i}}} {\text{max}}_{{\text{m}}} } \right|{\text{A}}_{{\text{m}}} - {\text{B}}_{{{\text{im}}}} } \right)} {\left| {{\text{A}}_{{{\text{m}} - }} {\text{B}}_{{{\text{im}}}} } \right|\left| { + \rho {\text{max}}_{{\text{i}}} {\text{max}}_{{\text{m}}} } \right|{\text{A}}_{{\text{m}}} - {\text{B}}_{{{\text{im}}}} |}}} \right. \kern-0pt}( {\left| {{\text{A}}_{{{\text{m}} - }} {\text{B}}_{{{\text{im}}}} } \right|\left| { + \rho {\text{max}}_{{\text{i}}} {\text{max}}_{{\text{m}}} } \right|{\text{A}}_{{\text{m}}} - {\text{B}}_{{{\text{im}}}} |}})$$

where εi is the correlation degree, A is the DRI, B is the evaluation vector, miniminm∣Am-Bim∣ is the second-level minimum difference, and maximaxm∣Am-Bim∣ is the second-level maximum difference; ρis the resolution coefficient, with the value range of 0–1, and *ρ* = 0. 5 was taken in this experiment.

$$\mathrm{DRI}(\mathrm{\%})=\frac{\mathrm{Measured\; values \;of\; each\; indicator\; in\; the\; treatment \;group}}{\mathrm{Measured \;values\; of\; indicators\; in\; the\; control\; group}}*100\mathrm{\%}$$

Affiliation value

When the indicator drought tolerance coefficient is positively correlated with drought resistance,

$$u(xi) = \frac{{xi_{j} - xi_{\min } }}{{xi_{\max } - xi_{\min } }}.$$

When the indicator drought tolerance coefficient is negatively correlated with drought resistance,

$$u(xi) = 1 - \frac{{xi_{j} - xi_{\min } }}{{xi_{\max } - xi_{\min } }}.$$

Drought Composite Score $D = \frac{1}{n}\sum\nolimits_{i = 1}^{n} {u(xi} )$.

## Results

### DRI Grading for *G. sinensis*

Indices of drought resistance the 58 *G. sinensis* families ranged from 0.44 to 1. Indices of drought resistance in the 58 *G. sinensis* families were clustered according to the Euclidean distance, and Euclidean distance was set 0.2, they could be classified into five clusters, namely, 8 HS families, 13 LS families, 12 LR families, 14 MR families, and 11 HR families type families (Table 2).

**Table 2:** Classification of DRI of *G. sinensis.*

Grading	Family Number	Number of families	DRI
HS	3, 8, 12, 31, 35, 46, 50, 52	8	0.52
LS	1, 5, 9, 10, 16,18, 25, 28, 29, 30, 32, 38, 42	13	0.7
LR	4, 6, 14, 19, 20, 22, 23, 26, 34, 43, 47, 55	12	0.78
MR	7, 21, 24, 27, 36, 37, 39, 41, 45, 49, 54, 56, 57, 58	14	0.86
HR	2, 11, 13, 15, 17, 33, 40, 44, 48, 51, 53	11	0.95

### Correlation analysis was conducted on the values of drought affiliation, photosynthetic physiological indexes, and drought resistance across various drought-resistant families of *G. sinensis*

The DRI was computed statistically by analysing the differences in morphological data of the seedling leaves when exposed to drought stress. It was observed that there were noteworthy variations in the DRI among different families, thus permitting a more precise assessment of drought resistance among various families (“Appendix 2”). Indices of drought resistance and affiliation function analysis of growth and photosynthetic physiological indices of each *G. sinensis* family was combined to analyse the five drought-resistant populations. The study extensively screened representative families for drought physiological indices in *G. sinensis*. These included HR representative family 17, MR representative family 49, LR representative family 23, LS representative family 28, and HS representative family 50. A comparison of the affiliation values for growth and photosynthetic physiological indices of the five selected families demonstrated notable variability in the affiliation values between them (“Appendix 2”). The values for seedling height, ground diameter, SPAD, Pn, Cs, Ci and Tr were 0.38, 0.32, 0.73, 0.63, 0.51, 0.11 and 0.62 greater in HR family than in HS family, indicating the potential of these indicators to identify drought resistance across different families of seedlings.

Indices of drought resistance of *G. sinensis* seedlings displayed a high correlation with growth and photosynthetic physiological indicators. Among the indicators, seedling height had the highest correlation with the drought tolerance index at 0.80 (Table 3). The correlation coefficients between SPAD value, ground diameter, and Pn, and DRI were 0.79, 0.60, and 0.66, respectively. In addition, the correlation coefficients of Cs and Tr with the drought tolerance index of *G. sinensis* were 0.63, whereas the correlation coefficient of Ci with the DRI was only 0.58. The DRI of *G. sinensis* exhibited a strong correlation with the height of the seedlings, SPAD values, and Tr index. By considering these factors, the differences in drought tolerance among families could be more accurately evaluated. Six indicators displayed correlations greater than 0.6 under severe stress, and each of these indicators is suitable for use as a reference for evaluating drought tolerance. While the correlation between photosynthesis physiological index and DRI was relatively high, it might be challenging to accurately assess drought tolerance among seedlings from different families only using photosynthesis physiological index, further researches will be necessary to investigate. When assessing the drought tolerance of seedlings from various families, the key indicators should be growth and leaf morphology indexes.

**Table 3:** Correlation coefficient, correlation degree and correlation order between drought resistance of *G. sinensis* and various indexes.

	HR	MR	LR	LS	HS	Degree	Order
Seedling height	0.65	1.00	0.69	0.81	0.88	0.80	1
Diameter	0.82	0.51	0.57	0.83	0.61	0.67	3
SPAD	0.88	0.84	0.98	0.61	0.64	0.79	2
Pn	0.75	0.61	0.97	0.61	0.38	0.66	4
Cs	0.56	0.55	0.83	0.85	0.33	0.63	6
C_i_	0.40	0.78	0.68	0.65	0.37	0.58	7
Tr	0.57	0.56	0.91	0.76	0.34	0.63	5

### Analysis of changes in physiological indicators of *G. sinensis* under drought stress

#### Changes in SS and SP content of *G. sinensis* under drought stress

**Figure 1:**
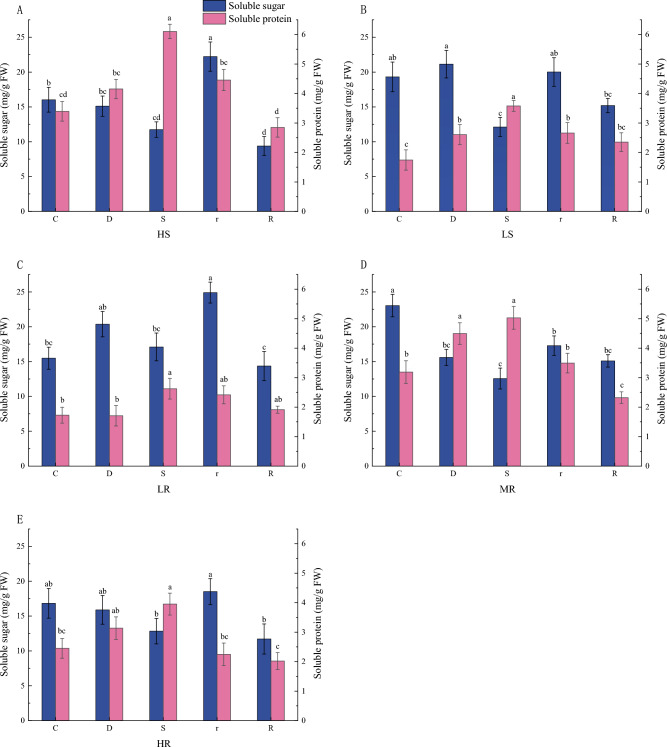
Changes of SS and SP contents in leaves of *G. sinensis* under drought stress and rehydration. *Note*: C, D, S, R and R represent control, natural drought for 7 d, natural drought for 14 d, rehydration for 1 d and rehydration for 3 d respectively. Different letters indicate significant differences at the *p* < 0.05 level, the same as below.

SS and SP are important osmoregulators. The SS content in the leaves of *G. sinensis* of all families showed less change under mild drought stress compared to the control, while the SS in the leaves of *G. sinensis* of HS, LS and MR families was significantly reduced under severe drought stress (Fig. 1). In contrast to the respective severe drought, SS content increased significantly at 1 d of rehydration and decreased significantly after 3 d of rehydration. There were no significant changes in SP in the HS, LR and HR family and a significant increase in SP in the LS and MR family under mild drought compared to their respective controls, however, SP were significantly increased under severe drought, and a significant decrease in SP was observed at 1 d of rehydration (except for LR family), as compared to their respective severe droughts.

#### Changes in Pro and MDA content of *G. sinensis* under drought stress

**Figure 2:**
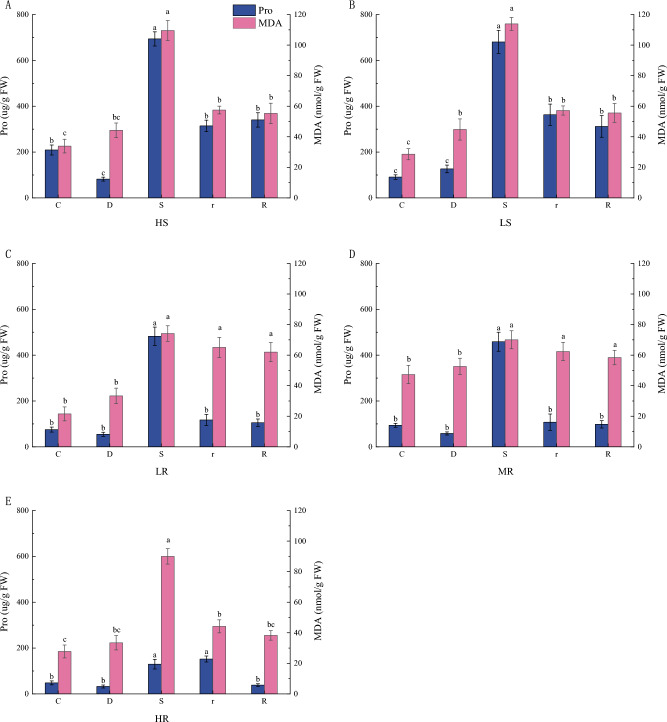
Changes of Pro and MDA content in leaves of *G. sinensis* under drought stress and rehydration.

The Pro in the leaves of *G. sinensis* was significantly reduced in the HS families during mild drought stress compared to the control, while the rest of the families showed insignificant changes (Fig. 2). At severe drought, Pro content was significantly increased in all the families. Leaf Pro content of HS, LS, LR and MR families were significantly reduced after rehydration compared to severe drought, while there was no significant change in leaf Pro content of HR family. MDA content in *G. sinensis* leaves increased during mild drought compared to their respective controls. The MDA content in all the *G. sinensis* leaves was significantly increased during severe drought (Fig. 2), indicating elevated ROS damage in *G. sinensis* leaves during severe drought. MDA in rehydration *G. sinensis* leaves was significantly reduced in HS, LS and HR family, and insignificantly changed in LR and MR family as compared to their respective severe drought.

#### Changes in antioxidant enzyme activities of *G. sinensis* under drought stress

**Figure 3:**
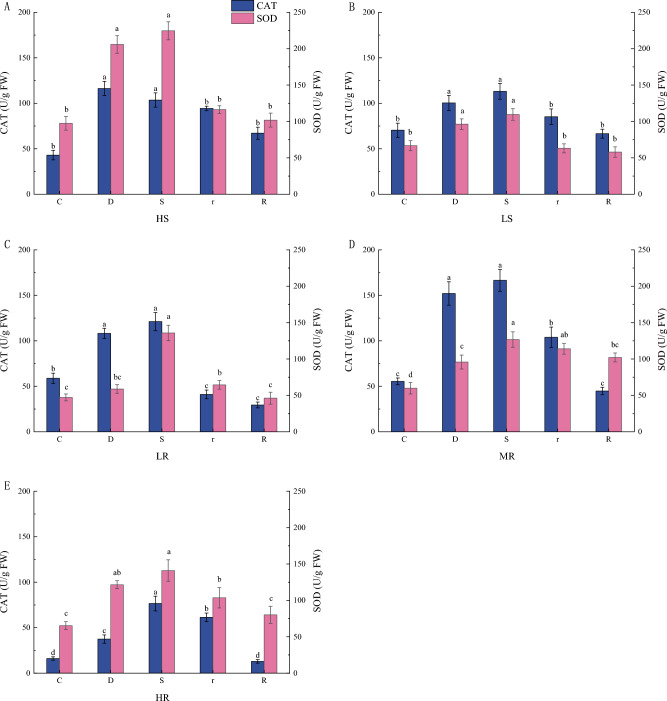
Changes of CAT and SOD enzyme activities in *G. sinensis* leaves under drought stress and rehydration.

CAT and SOD enzymes decompose H_2_O_2_ and O^2−^ in plant cells into H_2_O and O_2_, respectively, with the aim of reducing the buildup of intracellular ROS. This protects the integrity of organelles and cell membranes, maintaining the normal physiological functions of the cells. Both CAT and SOD activities within the leaves of *G. sinensis* were significantly increased under mild drought stress compared to their respective controls (Fig. 3). CAT activity within the *G. sinensis* leaves was significantly lower after rehydration compared to the respective severe drought stress. SOD enzyme activity was significantly higher in both LR and HR families during severe drought compared to mild drought. After rehydration, SOD activity was basically decreased in all families compared to severe drought.

**Figure 4:**
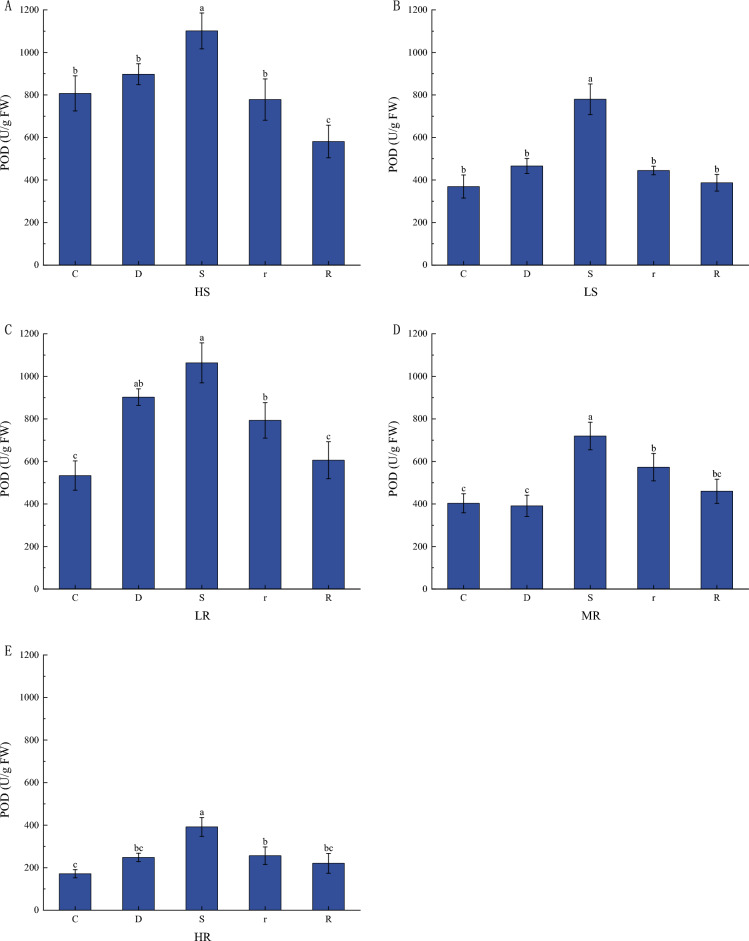
Changes of POD enzyme activity in *G. sinensis* leaves under drought stress and repeated rehydration.

POD activity was elevated but not significant under mild drought stress compared to their respective controls (except for the LS family). POD activity was significantly elevated in all the *G. sinensis* leaves under severe drought stress (Fig. 4). POD activity in *G. sinensis* leaves was significantly lower after 1 d of rehydration when compared to their respective severe drought stresses. The POD activity of the HR families was lower than the other four treatments throughout the drought-rehydration process.

### PCA of physiological indicators of drought tolerance in *G. sinensis*

**Figure 5:**
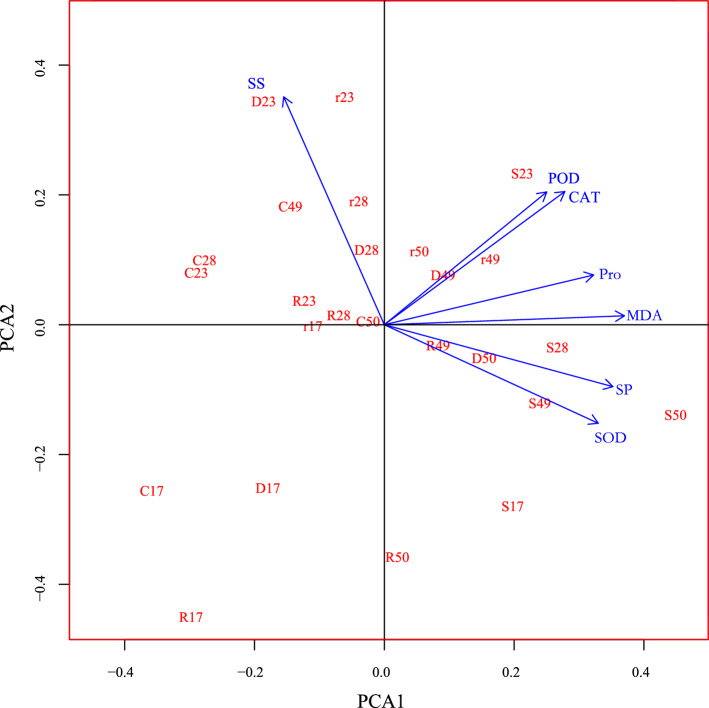
PCA of physiological characteristics of *G. sinensis* under drought stress and rehydration.

PCA is a multivariate statistical analysis method that reduces multiple variables to a small number of independent variables by reducing the dimensionality while retaining as much of the original information as possible. As shown in Fig. 5, for the physiological indicators of drought tolerance in *G. sinensis,* the first four principal components had a cumulative contribution rate of 85%, which could be used to represent the original data, and thus four principal components could be used to represent the seven physiological indicators of drought tolerance in *G. sinensis*. MDA, SS, Pro and CAT activity were the largest factor loadings in principal components 1, 2, 3 and 4, respectively, so these four indicators could be used as the main drought assessment indicators when *G. sinensis* were subjected to drought stress.

## Discussion

Plants may encounter recurrent droughts throughout their growth cycle. Drought is among the most pronounced abiotic stresses that restrict plant survival, growth and dispersion, impacting physiological and biochemical processes including photosynthesis, respiration, antioxidant activity, nutrient transport, and hormone homeostasis^30,31^. Plants’ ability to withstand drought is evident in various areas, such as growth and photosynthetic characteristics, anatomy, physiology and biochemistry. Several indicators for each area can be used to comprehensively evaluate drought tolerance, revealing how plants' response to drought stress involves synergistic and comprehensive multi-indicators^32^. The selection criteria for resistant materials typically include growth, physiological, and biochemical indexes. Combining multiple indexes aids in identifying resistant materials^33^. The seedling stage is a critical period in the plant life cycle and the most sensitive to adversity. The impact of drought stress on plant morphology renders the external features of plants a crucial and evident determinant of drought tolerance. Therefore, the assessment of plant tolerance to dry conditions can be facilitated by examining their external morphology^34^. Leaves of *G. sinensis* seedlings under drought stress in this study turned yellow in colour, curled and crumpled, wilted and fell off, and even the plants died, which is in agreement with the findings of various plants such as Citrus aurantium^35^. Leaf drooping, wilting and abscission may be the result of water deficiency leading to imbalance of water levels in the plant and decrease in cellular water potential and expansion pressure. Changes in leaf morphology can serve as a significant indicator for assessing the drought tolerance of *G. sinensis*. Differences in DRI indices among various families of *G. sinensis* were observed in this study. This finding is consistent with the results from previous research on evaluating drought tolerance in different watermelon varieties^36^. Our findings suggested that morphological indices played a crucial role in studying plant drought tolerance and could be utilised for screening drought-tolerant materials.

The correlation between the photosynthesis physiological index and DRI could indicate their proximity under drought stress. The greater the correlation, the greater the impact of the index on drought tolerance. In this study, it was found that a single photosynthetic index was inadequate to accurately assess the drought tolerance disparities among various *G. sinensis* families. A more precise drought tolerance identification could only be achieved through a meticulous screening of indexes with a high correlation to drought tolerance and comprehensive evaluation. Enhanced photosynthetic pigment content boosted the plant's photosynthetic capacity and improved water use efficiency. The correlation between SPAD value and DRI was highest at 0.79, indicating significant variation in photosynthetic pigment content changes across distinct drought-resistant families and periods. Consequently, SPAD value is a more suitable gauge for drought resistance assessment of *G. sinensis* seedlings. When *G. sinensis* were exposed to drought stress, the Pn, Cs, and Tr of their leaves decreased, demonstrating that photosynthesis was impacted. Ci had the smallest correlation with the DRI, and its variation was impacted by numerous factors, including light intensity and temperature. When there was enough light, photosynthesis was able to restore the used-up CO_2_, thus sustaining a high Ci with minimal variation. This could be the reason why the Ci correlation was the poorest and did not precisely demonstrate the drought resistance of *G. sinensis*. When assessing the drought resistance of *G. sinensis* seedlings using photosynthetic physiological indicators, it is recommended that SPAD and Pn should be chosen as primary indicators, other indicators should be evaluated comprehensively. The Chl content within maize leaves reduced during drought stress^17^, indicating reduced drought resistance. These findings align with the physiological indicator utilisation of Chl content for drought resistance identification in maize. During drought stress, *G. sinensis* closed its stomata and reduced transpiration to prevent excessive water loss. The reduction in leaf water content led to an increase in the relative concentration of Chl. Meanwhile, severe drought stress hindered Chl synthesis and hastened Chl degradation^37^. Therefore, the chl content can serve as a metric to assess the drought resistance of various plants, subject to distinct gradations of drought. Drought conditions stunted the growth of *Artemisia selengensis* by inhibiting plant height, biomass^38^, and Chl content. Additionally, this study found drought to have an inhibitory effect on the growth of seedling height and ground diameter, as well as biomass of *G. sinensis* seedlings, which may be attributed to photosynthesis being severely inhibited.

The capacity of plants to endure harsh surroundings is closely correlated with their antioxidant and osmoregulatory capabilities^39^. Under conditions of drought stress, plants generate a substantial quantity of ROS and engage the mechanism of ROS scavenging. The amount and speed of ROS buildup have a positive correlation with the severity of drought experienced by plants^40^. High levels of ROS resulted in significant membrane lipid peroxidation leading to oxidative harm to proteins and cell membranes, leading to the creation of high levels  of MDA^41^. MDA is the final result of the peroxidation of membrane lipids, which can induce denaturation of proteins and nucleic acids^42^. The content of MDA can indicate the extent of harm exerted on plant cells by drought stress and serve as a crucial indicator to assess the drought tolerance among plants. In this study, it was observed that there was a slight rise in MDA levels during mild drought, suggesting that *G. sinensis* seedlings were less affected by the drought. However, in severe drought, MDA levels in *G. sinensis* increased sharply among five families. There was a reduction in MDA following rehydration, which suggested that rehydration mitigated membrane lipid peroxidation and membrane permeability. To mitigate the harm caused by ROS, cells activated their antioxidant enzyme mechanism, thereby establishing a close correlation between the antioxidant enzyme response system and drought resistance in plants. ROS  first reacted with SOD to produce low-oxidizing H_2_O_2_. Subsequently, hydrogen peroxide would undergo further catalytic reactions with POD and CAT, resulting in its breakdown into H_2_O_2_ and O_2_^43^. In mild drought, *G. sinensis* was less damaged and the activities of SOD and CAT were significantly increased, indicating that in mild drought, the enzymes SOD and CAT scavenge excess ROS^40^, which played an important role in the resistance of *G. sinensis* seedlings to mild drought, whereas in severe drought, the enzyme activities were increased, which was associated with the drastic increase in ROS during severe drought. The different increases in antioxidant enzyme activities under drought stress indicated that the mechanisms of ROS scavenging in *G. sinensis* from different families were not identical and that the antioxidant enzyme activities at different drought and rehydration stages were somewhat specific. The decrease in antioxidant enzyme activities after rehydration indicated that the damage caused by drought stress to the seedlings was reduced after rehydration. Chl content, MDA content, Pro content, SOD, CAT and POD activities could be used as physiological indicators for the identification of drought tolerance in maize^17^, watermelon^44^ and *Phaseolus vulgaris* L^32^. Photosynthesis indices, leaf thickness, biomass, plant height, and leaf pro content can serve as indicators for identifying drought tolerance in *Camellia oleifera* seedlings^45^and are similar to the findings of our study. The activities of protective enzymes under drought stress were the primary indicators for evaluating the drought tolerance of *Platanus* seedlings^46^. Pro content was its secondary indicator, relative water content and MDA content were the secondary indicators. The PCA demonstrated that MDA was the primary physiological index for assessing drought resistance in *G. sinensis* seedlings. SS, SP, Pro content, and antioxidant enzyme activity were recommended as additional parameters. The variability in the degree of change and evaluation capacity of each index under drought stress was found to differ between tree species. The aforementioned physiological indices can be used as evaluative measures to a certain extent in order to reflect the drought resistance of plants.

Osmoregulation is a crucial adaptation mechanism for plants dealing with drought. They actively regulate their cell's osmotic pressure to increase water absorption. As a result, they accumulate significant amounts of osmoregulatory substances. SS, SP, and Pro are key osmoregulatory substances that play a pivotal role in regulating osmotic pressure, stabilising protein and cell membrane structures, and scavenging excess free radicals^47–49^. Studies on *Hemerocallis*^50^ and *Carex breviculmis*^51^ revealed a noteworthy augmentation in SP content during moderate drought and a reduction in SS content during severe and moderate drought conditions. Pro enhanced the drought tolerance of *fennel*^52^. Furthermore, there was a significant increase in the accumulation of Pro and sugar in strawberry^53^ leaves under drought stress compared to the control. SP, Pro and SS were used as important evaluation indicators for selecting drought tolerant varieties and materials^54^. It was discovered through this study that SP and Pro increased of *G. sinensis* leaves, elevating the osmotic potential under drought conditions. In contrast, SS levels sharply decreased similarly to previous research findings. The reduction in SS content could be attributed to the suppression of SS synthesis, as the drought stress led to a decrease in photosynthesis in the plants. Even after rehydration, the SS content remained lower than that of the control, suggesting that the damage caused by drought is challenging to mitigate in a brief period. Drought stress hindered the synthesis of protein, leading to reduced SP content in plants^55^. During the severe drought in the current investigation, the Pro concentration of *G. sinensis* significantly rose in all five families. It subsequently decreased partially after rehydration, which could be attributed to the Pro transforming into glutamate via Pro dehydrogenase (*PDH*) and *P5C dehydrogenase* (*P5CDH*)^56^. Pro serves as the primary osmotic regulator in *G. sinensis* experiencing severe drought and is a crucial indicator for assessing drought tolerance^57^.

Drought tolerance in plants is a multifaceted biological characteristic controlled by numerous genes and metabolic pathways. A solitary indicator often fails to accurately assess the extent of a plant's resistance to drought. Therefore, it becomes essential to conduct a comprehensive evaluation of multiple indicators, which necessitates a scientific evaluation system^58^. Leaf morphological indicators, seedling height, SPAD value, Pn, and MDA can serve as dependable predictors in assessing the drought tolerance of *G. sinensis* seedlings. The use of photosynthesis and other physiological indicators for the evaluation of drought tolerance in *G. sinensis* seedlings should be analysed in a comprehensive manner using multiple indicators and multiple methods. Scholars both domestically and internationally have progressively employed methods such as drought tolerance coefficient, DRI, PCA, affiliation function analysis, factor analysis, regression analysis, grey correlation degree analysis, and cluster analysis in combination to thoroughly appraise the drought tolerance of various plant species^59^. This ensures that the outcomes are more impartial and rational.

## Conclusion

In this study, 58 seedlings of *G. sinensis* families were subjected to drought stress and rehydration treatment and the DRI was used to classify the seedling families of *G. sinensis* into five groups: HS, LS, LR, MR and HR families. The DRI was used as the main index, combined with growth and leaf morphology and photosynthetic physiological indexes to comprehensively screen five representative families of different drought tolerance families. The Pro, SP, and MDA contents, along with the CAT, POD, and SOD activities of the seedlings from the five families, increased with the drought's intensity. The highest values were primarily observed during the severe drought period and gradually decreased after rehydration. SP, Pro and MDA contents, CAT, POD and SOD activities of the HR families were 1.70, 0.61, 2.24, 3.75, 1.28 and 1.16 times higher than those of the natural control treatments during the severe drought period. The SS content decreased with the duration of the drought and reached its peak after 1 d of rehydration. PCA identified MDA and SS as the primary indicators of response to both drought and rehydration within *G. sinensis*. Changes in osmoregulatory substance content and increased antioxidant enzyme activity could be essential factors in the response of *G. sinensis* to drought mechanisms. Leaf morphological indicators, seedling height, SPAD value, Pn, and MDA are reliable indicators for evaluating the drought tolerance of *G. sinensis* seedlings. When assessing the drought resistance of *G. sinensis* seedlings through photosynthesis and other physiological indicators, it is necessary to utilise multiple indicators and methods for an all-encompassing analysis. This evaluation methods can serve as a reference point in screening superior drought-resistant families of *G. sinensis*. *G. sinensis* is poorly drought tolerant during the seedling period, so drought tolerant families should be selected for production applications and adequate moisture should be ensured for the seedlings.

### Supplementary Information

Supplementary Information 1.

Supplementary Information 2.

Supplementary Information 3.

Supplementary Information 4.

## Data Availability

All data are presented in the article, and can be requested from the corresponding author if required.

## Abbreviations

CAT: Catalase
Chl: Chlorophyll
DRI: Drought Resistance Index
HR: High resistance
HS: Highly susceptible
Ci: Intercellular CO_2_ concentration
LR: Low resistance
LS: Low susceptibility
MDA: Malondialdehyde
MR: Medium resistance
Pn: Net photosynthetic rate
POD: Peroxidase
PCA: Principal component analysis
Pro: Proline
ROS: Reactive oxygen species
SS: Soluble sugars
SP: Soluble protein
SOD: Superoxide dismutase
SPAD: Soil plant analysis development
Cs: Stomatal conductance
Tr: Transpiration rate
