# Supplementary material for: Physiological response and drought resistance evaluation of Gleditsia sinensis seedlings under drought-rehydration state
Source: Sci Rep. 2023 Nov 15;13:19963. doi: 10.1038/s41598-023-45394-8 (PMC10651932; doi:10.1038/s41598-023-45394-8)
Supplement: Supplementary file 1 — Supplementary Information 1. [file 41598_2023_45394_MOESM1_ESM.docx]

**Appendix 1** *G. sinensis* seeds family Collection location, longitude and latitude and origin

| Family number | Collection location | Latitude and Longitude | Origin |
| --- | --- | --- | --- |
| 1 | Dafang County, Bijie City | 27.082391N,105.563625E | wildlife material |
| 2 | Huichuan District, Zunyi City | 27.724314N,106.712140E | wildlife material |
| 3 | Dafang County, Bijie City | 27.085352N,105.563985E | wildlife material |
| 4 | Changshun County, Qiannan Autonomous Prefecture | 26.031890N,106.507135E | wildlife material |
| 5 | Fuquan City, Qiannan Autonomous Prefecture | 26.747855N,107.632816E | wildlife material |
| 6 | Kaili City, Qiandongnan Autonomous Prefecture | 26.759472N,107.642094E | wildlife material |
| 7 | Kaili City, Qiandongnan Autonomous Prefecture | 26.645204N,108.053385E | wildlife material |
| 8 | Luodian County, Qiannan Autonomous Prefecture | 25.242420N,106.724355E | wildlife material |
| 9 | Duyun City, Qiannan Autonomous Prefecture | 26.170862N,107.620378E | wildlife material |
| 10 | Longli County, Qiannan Autonomous Prefecture | 26.462597N,106.936178E | wildlife material |
| 11 | Yunyan District, Guiyang City | 26.617124N,106.705100E | wildlife material |
| 12 | Jiangkou County, Tongren City | 27.713548N,108.852698E | wildlife material |
| 13 | Pingba District, Anshun City | 26.227840N,106.277749E | wildlife material |
| 14 | Sinan County, Tongren City | 27.840217N,108.007200E | wildlife material |
| 15 | Qingzhen City, Guiyang City | 26.577037N,106.524551E | wildlife material |
| 16 | Wanshan District, Tongren City | 26.577266N,106.520722E | wildlife material |
| 17 | Huaxi District, Guiyang City | 26.457633N,106.562547E | Artificially cultivated |
| 18 | Pingba District, Anshun City | 26.227840N,106.277731E | wildlife material |
| 19 | Nanming District, Guiyang City | 26.545881N,106.702472E | wildlife material |
| 20 | Qingzhen City, Guiyang City | 26.577266N,106.520722E | wildlife material |
| 21 | Qingzhen City, Guiyang City | 26.577266N,106.520132E | wildlife material |
| 22 | Dafang County, Bijie City | 27.072283N,105.570231E | wildlife material |
| 23 | Kaili City, Qiandongnan Autonomous Prefecture | 26.608297N,107.846001E | wildlife material |
| 24 | Kaili City, Qiandongnan Autonomous Prefecture | 26.670138N,108.114827E | wildlife material |
| 25 | Fuquan City, Qiannan Autonomous Prefecture | 26.689134N,107.516826E | wildlife material |
| 26 | Fuquan City, Qiannan Autonomous Prefecture | 26.723616N,107.496127E | wildlife material |
| 27 | Kaili City, Qiandongnan Autonomous Prefecture | 26.723867N,107.496048E | wildlife material |
| 28 | Huaxi District, Guiyang City | 26.457633N,106.562511E | Artificially cultivated |
| 29 | Kaili City, Qiandongnan Autonomous Prefecture | 26.645204N,108.053379E | wildlife material |
| 30 | Dushan County, Qiannan Autonomous Prefecture | 25.459770N,107.357480E | wildlife material |
| 31 | Dushan County, Qiannan Autonomous Prefecture | 25.794368N,107.538139E | wildlife material |
| 32 | Kaili City, Qiandongnan Autonomous Prefecture | 26.470455N,107.902907E | wildlife material |
| 33 | Huaxi District, Guiyang City | 26.457633N,106.562587E | Artificially cultivated |
| 34 | Fuquan City, Qiannan Autonomous Prefecture | 26.723561N,107.496182E | wildlife material |
| 35 | Huaxi District, Guiyang City | 26.457633N,106.562591E | Artificially cultivated |
| 36 | Dafang County, Bijie City | 27.072283N,105.570231E | wildlife material |
| 37 | Fuquan City, Qiannan Autonomous Prefecture | 26.723867N,107.496048E | wildlife material |
| 38 | Fuquan City, Qiannan Autonomous Prefecture | 26.470455N,107.902907E | wildlife material |
| 39 | Pingba District, Anshun City | 26.227841N,106.277789E | wildlife material |
| 40 | Dafang County, Bijie City | 27.072283N,105.570231E | wildlife material |
| 41 | Huaxi District, Guiyang City | 26.486303N,106.540659E | wildlife material |
| 42 | Duyun City, Qiannan Autonomous Prefecture | 26.253451N,107.306527E | wildlife material |
| 43 | Majiang County, Qiandongnan Autonomous Prefecture | 26.480722N,107.523016E | wildlife material |
| 44 | Liuzhi Special District, Liupanshui City | 26.207980N,105.565216E | wildlife material |
| 45 | Kaili City, Qiandongnan Autonomous Prefecture | 26.734093N,107.745529E | wildlife material |
| 46 | Dushan County, Qiannan Autonomous Prefecture | 25.794368N,107.538101E | wildlife material |
| 47 | Liuzhi Special District, Liupanshui City | 26.280854N,105.450931E | wildlife material |
| 48 | Kaili City, Qiandongnan Autonomous Prefecture | 26.599192N,107.767602E | wildlife material |
| 49 | Yunyan District, Guiyang City | 26.604321N,106.707620E | wildlife material |
| 50 | Yunyan District, Guiyang City | 26.617124N,106.705977E | wildlife material |
| 51 | Fuquan City, Qiannan Autonomous Prefecture | 26.762868N,107.638153E | wildlife material |
| 52 | Liuzhi Special District, Liupanshui City | 26.198520N,105.537491E | wildlife material |
| 53 | Sinan County, Tongren City | 26.198520N,105.537491E | wildlife material |
| 54 | Huaxi District, Guiyang City | 26.457633N,106.562502E | Artificially cultivated |
| 55 | Duyun City, Qiannan Autonomous Prefecture | 26.340831N,107.496766E | wildlife material |
| 56 | Anlong County, Qianxinan Autonomous Prefecture | 25.033313N,105.290145E | wildlife material |
| 57 | Anlong County, Qianxinan Autonomous Prefecture | 25.033549N,105.291572E | wildlife material |
| 58 | Ceheng County, Qianxinan Autonomous Prefecture | 24.910550N,105.976284E | wildlife material |
