# Supplementary material for: Physiological response and drought resistance evaluation of Gleditsia sinensis seedlings under drought-rehydration state
Source: Sci Rep. 2023 Nov 15;13:19963. doi: 10.1038/s41598-023-45394-8 (PMC10651932; doi:10.1038/s41598-023-45394-8)
Supplement: Supplementary file 2 — Supplementary Information 2. [file 41598_2023_45394_MOESM2_ESM.docx]

**Appendix 2** Drought resistance membership value and DRI of *G. sinensis* family

| **Grading** | | **Family number** | **Seedling height** | **Diameter** | **SPAD** | **Pn** | **Cs** | **Ci** | **Tr** | **Affiliation value** | **Rank** | **DRI** |
| --- | --- | --- | --- | --- | --- | --- | --- | --- | --- | --- | --- | --- |
| HR | | 17 | 0. 99 | 0. 97 | 1. 00 | 0. 99 | 0. 71 | 0. 52 | 0. 84 | 0. 86 | 1 | 1. 00 |
|  |  | 51 | 0. 90 | 0. 97 | 0. 12 | 0. 70 | 0. 58 | 0. 66 | 0. 66 | 0. 66 | 2 | 0. 94 |
|  |  | 15 | 1. 00 | 1. 00 | 0. 69 | 0. 59 | 0. 30 | 0. 55 | 0. 39 | 0. 65 | 3 | 0. 94 |
|  |  | 53 | 0. 84 | 0. 74 | 0. 75 | 0. 22 | 0. 20 | 0. 78 | 0. 22 | 0. 54 | 4 | 0. 94 |
|  |  | 33 | 0. 89 | 0. 67 | 0. 85 | 0. 49 | 0. 18 | 0. 11 | 0. 23 | 0. 49 | 5 | 0. 94 |
|  |  | 44 | 0. 91 | 0. 84 | 0. 00 | 0. 40 | 0. 33 | 0. 52 | 0. 38 | 0. 48 | 6 | 0. 94 |
|  |  | 48 | 0. 87 | 0. 73 | 0. 13 | 0. 39 | 0. 26 | 0. 58 | 0. 32 | 0. 47 | 7 | 1. 00 |
|  |  | 40 | 0. 78 | 0. 93 | 0. 29 | 0. 30 | 0. 17 | 0. 48 | 0. 22 | 0. 45 | 8 | 0. 94 |
|  |  | 2 | 0. 54 | 0. 91 | 0. 52 | 0. 28 | 0. 17 | 0. 44 | 0. 20 | 0. 44 | 9 | 0. 94 |
|  |  | 13 | 0. 42 | 0. 33 | 0. 65 | 0. 39 | 0. 15 | 0. 33 | 0. 20 | 0. 35 | 10 | 0. 94 |
|  |  | 11 | 0. 17 | 0. 24 | 0. 61 | 0. 29 | 0. 17 | 0. 58 | 0. 23 | 0. 33 | 11 | 0. 94 |
| MR | 45 | | 0. 98 | 0. 98 | 0. 48 | 0. 46 | 0. 28 | 0. 61 | 0. 38 | 0. 59 | 1 | 0. 89 |
|  | 56 | | 0. 39 | 0. 28 | 0. 46 | 0. 75 | 0. 66 | 0. 75 | 0. 75 | 0. 58 | 2 | 0. 83 |
|  | 57 | | 0. 79 | 0. 67 | 0. 79 | 0. 36 | 0. 27 | 0. 65 | 0. 34 | 0. 55 | 3 | 0. 83 |
|  | 36 | | 0. 71 | 0. 96 | 0. 65 | 0. 42 | 0. 23 | 0. 49 | 0. 29 | 0. 53 | 4 | 0. 83 |
|  | 37 | | 0. 95 | 0. 69 | 0. 54 | 0. 47 | 0. 19 | 0. 56 | 0. 27 | 0. 52 | 5 | 0. 89 |
|  | 7 | | 0. 81 | 0. 87 | 0. 44 | 0. 55 | 0. 24 | 0. 44 | 0. 31 | 0. 52 | 6 | 0. 89 |
|  | 49 | | 0. 84 | 0. 82 | 0. 40 | 0. 41 | 0. 21 | 0. 50 | 0. 28 | 0. 49 | 7 | 0. 83 |
|  | 39 | | 0. 67 | 0. 36 | 0. 96 | 0. 38 | 0. 23 | 0. 53 | 0. 29 | 0. 49 | 8 | 0. 83 |
|  | 41 | | 0. 74 | 0. 85 | 0. 36 | 0. 40 | 0. 20 | 0. 56 | 0. 25 | 0. 48 | 9 | 0. 83 |
|  | 24 | | 0. 94 | 0. 61 | 0. 59 | 0. 11 | 0. 12 | 0. 80 | 0. 15 | 0. 47 | 10 | 0. 83 |
|  | 27 | | 0. 93 | 0. 07 | 0. 32 | 0. 35 | 0. 32 | 0. 78 | 0. 41 | 0. 46 | 11 | 0. 89 |
|  | 21 | | 0. 83 | 0. 00 | 0. 62 | 0. 40 | 0. 28 | 0. 66 | 0. 34 | 0. 45 | 12 | 0. 89 |
|  | 54 | | 0. 98 | 0. 97 | 0. 08 | 0. 00 | 0. 00 | 0. 68 | 0. 00 | 0. 39 | 13 | 0. 83 |
|  | 58 | | 0. 29 | 0. 58 | 0. 46 | 0. 32 | 0. 23 | 0. 53 | 0. 27 | 0. 38 | 14 | 0. 89 |
| LR | 34 | | 0. 99 | 0. 72 | 0. 55 | 0. 86 | 0. 87 | 0. 91 | 1. 00 | 0. 84 | 1 | 0. 78 |
|  | 22 | | 0. 94 | 0. 89 | 0. 05 | 0. 55 | 0. 49 | 0. 64 | 0. 59 | 0. 59 | 2 | 0. 78 |
|  | 19 | | 0. 57 | 0. 77 | 0. 93 | 0. 39 | 0. 29 | 0. 61 | 0. 34 | 0. 56 | 3 | 0. 78 |
|  | 4 | | 0. 94 | 0. 80 | 0. 83 | 0. 53 | 0. 25 | 0. 17 | 0. 32 | 0. 55 | 4 | 0. 78 |
|  | 6 | | 0. 80 | 0. 88 | 0. 58 | 0. 55 | 0. 25 | 0. 44 | 0. 30 | 0. 54 | 5 | 0. 78 |
|  | 23 | | 0. 83 | 0. 74 | 0. 61 | 0. 72 | 0. 33 | 0. 00 | 0. 45 | 0. 53 | 6 | 0. 78 |
|  | 14 | | 0. 98 | 0. 75 | 0. 31 | 0. 29 | 0. 20 | 0. 57 | 0. 26 | 0. 48 | 7 | 0. 78 |
|  | 20 | | 0. 94 | 0. 93 | 0. 30 | 0. 27 | 0. 15 | 0. 53 | 0. 19 | 0. 47 | 8 | 0. 78 |
|  | 55 | | 0. 68 | 0. 79 | 0. 44 | 0. 12 | 0. 16 | 0. 87 | 0. 20 | 0. 47 | 9 | 0. 78 |
|  | 47 | | 0. 72 | 0. 57 | 0. 50 | 0. 33 | 0. 20 | 0. 61 | 0. 27 | 0. 46 | 10 | 0. 78 |
|  | 26 | | 0. 78 | 0. 85 | 0. 21 | 0. 24 | 0. 16 | 0. 63 | 0. 20 | 0. 44 | 11 | 0. 78 |
|  | 43 | | 0. 38 | 0. 90 | 0. 23 | 0. 20 | 0. 09 | 0. 66 | 0. 12 | 0. 37 | 12 | 0. 78 |
| LS | 30 | | 0. 87 | 0. 82 | 0. 66 | 0. 29 | 0. 35 | 1. 00 | 0. 32 | 0. 62 | 1 | 0. 72 |
|  | 29 | | 0. 87 | 0. 95 | 0. 41 | 0. 49 | 0. 46 | 0. 46 | 0. 51 | 0. 59 | 2 | 0. 67 |
|  | 16 | | 0. 83 | 0. 71 | 0. 44 | 0. 51 | 0. 43 | 0. 72 | 0. 51 | 0. 59 | 3 | 0. 67 |
|  | 5 | | 0. 70 | 0. 83 | 0. 42 | 0. 43 | 0. 26 | 0. 50 | 0. 32 | 0. 49 | 4 | 0. 72 |
|  | 1 | | 0. 86 | 0. 80 | 0. 46 | 0. 30 | 0. 21 | 0. 55 | 0. 23 | 0. 49 | 5 | 0. 72 |
|  | 25 | | 0. 70 | 0. 55 | 0. 23 | 0. 43 | 0. 35 | 0. 67 | 0. 41 | 0. 48 | 6 | 0. 67 |
|  | 10 | | 0. 99 | 0. 66 | 0. 66 | 0. 20 | 0. 09 | 0. 37 | 0. 13 | 0. 44 | 7 | 0. 72 |
|  | 42 | | 0. 78 | 0. 75 | 0. 30 | 0. 32 | 0. 19 | 0. 36 | 0. 24 | 0. 42 | 8 | 0. 67 |
|  | 28 | | 0. 76 | 0. 79 | 0. 26 | 0. 02 | 0. 08 | 0. 89 | 0. 10 | 0. 41 | 9 | 0. 72 |
|  | 32 | | 0. 96 | 0. 58 | 0. 08 | 0. 18 | 0. 16 | 0. 67 | 0. 18 | 0. 40 | 10 | 0. 72 |
|  | 9 | | 0. 45 | 0. 17 | 0. 15 | 0. 26 | 0. 24 | 0. 88 | 0. 29 | 0. 35 | 11 | 0. 67 |
|  | 38 | | 0. 00 | 0. 02 | 0. 43 | 0. 48 | 0. 35 | 0. 71 | 0. 41 | 0. 34 | 12 | 0. 72 |
|  | 18 | | 0. 53 | 0. 58 | 0. 36 | 0. 17 | 0. 09 | 0. 48 | 0. 12 | 0. 33 | 13 | 0. 72 |
| HS | 31 | | 0. 96 | 0. 97 | 0. 49 | 0. 73 | 0. 85 | 0. 89 | 0. 94 | 0. 83 | 1 | 0. 61 |
|  | 12 | | 0. 32 | 0. 57 | 0. 30 | 1. 00 | 1. 00 | 1. 00 | 0. 94 | 0. 73 | 2 | 0. 50 |
|  | 8 | | 0. 86 | 0. 85 | 0. 63 | 0. 65 | 0. 37 | 0. 50 | 0. 42 | 0. 61 | 3 | 0. 50 |
|  | 52 | | 0. 34 | 0. 75 | 0. 56 | 0. 46 | 0. 45 | 0. 80 | 0. 49 | 0. 55 | 4 | 0. 56 |
|  | 35 | | 0. 71 | 0. 87 | 0. 60 | 0. 29 | 0. 24 | 0. 82 | 0. 28 | 0. 54 | 5 | 0. 50 |
|  | 46 | | 0. 12 | 0. 28 | 0. 35 | 0. 46 | 0. 56 | 0. 95 | 0. 69 | 0. 49 | 6 | 0. 56 |
|  | 3 | | 0. 77 | 0. 79 | 0. 30 | 0. 21 | 0. 15 | 0. 62 | 0. 19 | 0. 43 | 7 | 0. 56 |
|  | 50 | | 0. 61 | 0. 65 | 0. 27 | 0. 36 | 0. 20 | 0. 41 | 0. 22 | 0. 39 | 8 | 0. 44 |
