# Supplementary material for: Physiological response and drought resistance evaluation of Gleditsia sinensis seedlings under drought-rehydration state
Source: Sci Rep. 2023 Nov 15;13:19963. doi: 10.1038/s41598-023-45394-8 (PMC10651932; doi:10.1038/s41598-023-45394-8)
Supplement: Supplementary file 3 — Supplementary Information 3. [file 41598_2023_45394_MOESM3_ESM.docx]

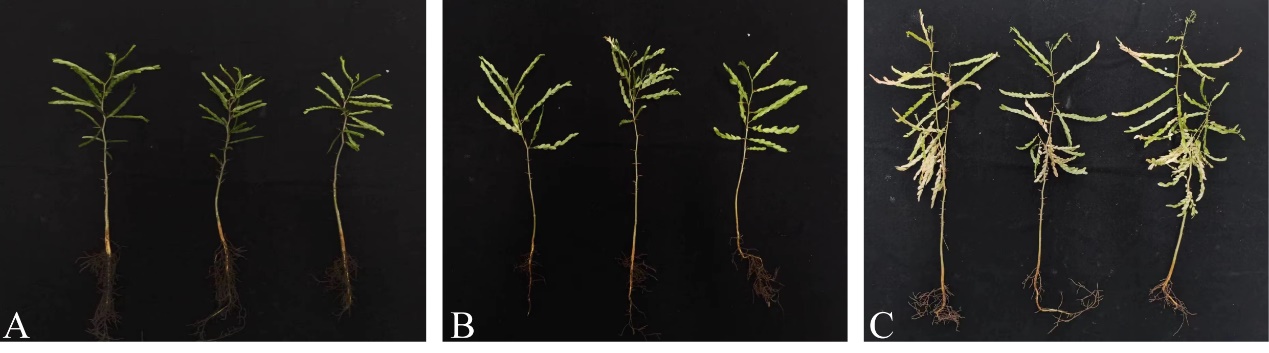


Figure 6 Morphological pictures of *Gleditsia sinensis* seedlings with different drought levels.

Note: A, control; B, mild drought; C, severe drought.
