# Supplementary figures and images for: Physiological response and drought resistance evaluation of Gleditsia sinensis seedlings under drought-rehydration state
Source: Sci Rep. 2023 Nov 15;13:19963. doi: 10.1038/s41598-023-45394-8 (PMC10651932; doi:10.1038/s41598-023-45394-8)

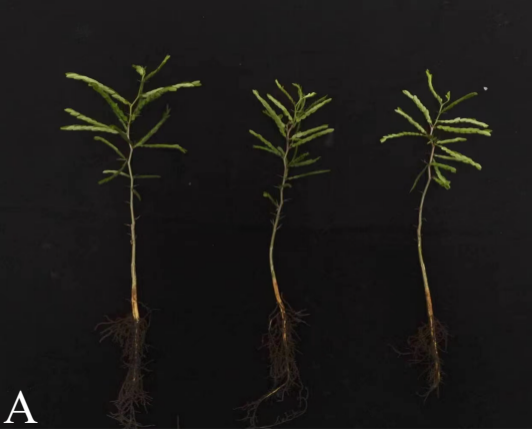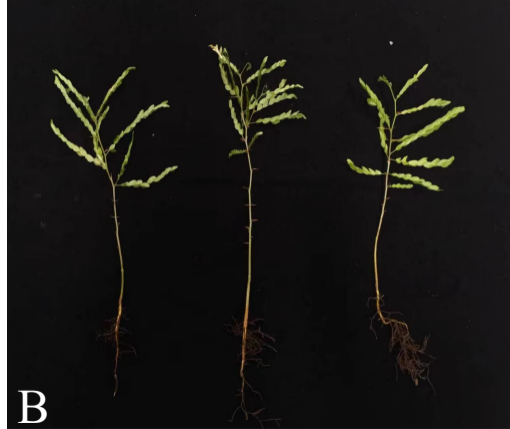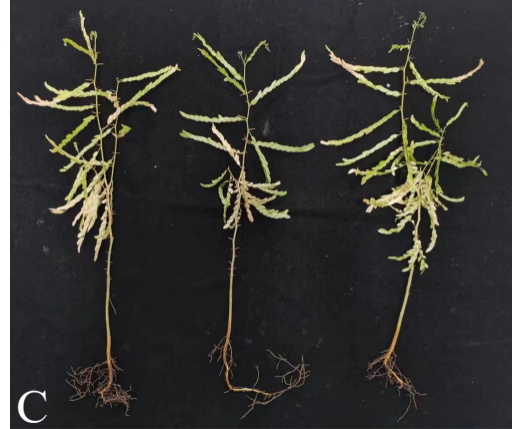

Supplement: Supplementary file 4 — Supplementary Information 4. [file 41598_2023_45394_MOESM4_ESM.pdf]
